# New *CDH3* mutation in the first Spanish case of hypotrichosis with juvenile macular dystrophy, a case report

**DOI:** 10.1186/s12881-016-0364-5

**Published:** 2017-01-07

**Authors:** Fiona Blanco-Kelly, Luciana Rodrigues-Jacy da Silva, Iker Sanchez-Navarro, Rosa Riveiro-Alvarez, Miguel Angel Lopez-Martinez, Marta Corton, Carmen Ayuso

**Affiliations:** 1 Department of Medical Genetics, Instituto de Investigación Sanitaria - Fundación Jiménez Díaz, (IIS-FJD, UAM), Madrid, Spain; 2 Centro de Investigaciones Biomédicas en Red de Enfermedades Raras (CIBERER), Instituto Carlos IIII (ISCIII), Madrid, Spain; 3 Department of Genomics and Genetics, Fundación Jiménez Díaz University Hospital, Av. Reyes Católicos n° 2, 28040 Madrid, Spain

**Keywords:** Macular dystrophy, *CDH3*, Hypotrichosis, Syndromic retinal dystrophy, Case report

## Abstract

**Background:**

*CDH3* on 16q22.1 is responsible for two rare autosomal recessive disorders with hypotrichosis and progressive macular dystrophy: Hypotrichosis with Juvenile Macular Dystrophy and Ectodermal Dysplasia, Ectrodactyly and Macular Dystrophy. We present a new case of Hypotrichosis with Juvenile Macular Dystrophy.

**Case presentation:**

A Spanish male born in 1998 from non-consanguineous healthy parents with a suspected diagnosis of Keratosis Follicularis Spinulosa Decalvans and Retinitis Pigmentosa Inversa referred to our Genetics Department (IIS-Fundación Jiménez Díaz).

Molecular study of *ABCA4* was performed, and a heterozygous missense p.Val2050Leu variant in *ABCA4* was found.

Clinical revision reclassified this patient as Hypotrichosis with Juvenile Macular Dystrophy. Therefore, further *CDH3* sequencing was performed showing a novel maternal missense change p.Val205Met (probably pathogenic by *in silico* analysis), and a previously reported paternal frameshift c.830del;p.Gly277Alafs*20, thus supporting the clinical diagnosis..

**Conclusions:**

This is not only the first Spanish case with this clinical and molecular diagnosis, but a new mutation has been described in *CDH3*. Moreover, this work reflects the importance of joint assessment of clinical signs and evaluation of pedigree for a correct genetic study approach and diagnostic.

## Background

The *CDH3* gene, on16q22.1, encodes for P-cadherin, which is expressed in retina epithelial cells and in hair follicles, being responsible for adherens junctions (calcium dependent cell-cell adhesion molecule) in these and other epithelial tissues [1, 2]. Additionally, *CDH3* is thought to be a marker of the epithelial tissues progenitor cells and it has been found to be expressed in human embryonic stem cells [3, 4].

This gene is responsible for two rare autosomal recessive disorders: Hypotrichosis with Juvenile Macular Dystrophy (HJMD, OMIM: 601553) [5–17] and Ectodermal Dysplasia, Ectrodactyly and Macular Dystrophy (EEM, OMIM: 225280) [18]. Both disorders characterized by hypotrichosis and progressive retinal dystrophy.

To date, according to Orphanet (http://www.orpha.net, date of access 11/05/2016) the frequencies of HJMD and EEM are unknown and across the world only 50 cases of HJMD and approximately 15 cases of EEM (only three with molecular characterization) [8, 19] have been described.

HJMD has been described as macular degeneration and short sparse scalp hair from birth with hair loss during the first months of life [5]. The retinal phenotype is nowadays being re-evaluated. It has been suggested that earlier onset is more likely than the juvenile stated on its name [7, 20–22], and that there is a wider involvement of the retina (not only the macula) [7, 20–23]. Thus, defining the retinal alteration as a more complex retinal dystrophy rather than a solely macular dystrophy.

The hair can present different types of abnormalities being pili-torti one of the most frequent (twisted hair shafts on the long axis, which are brittle, and break at varying lengths with many areas appearing bald) [5, 24]. It usually affects the scalp but can also affect eyebrows and eyelashes (body hair is normal) [5, 24].

Patients with EEM syndrome, additionally exhibit a wider range of features such as split hand/foot malformation (SHFM) or ectrodactyly along or not with oligodontia, enamel hypoplasia, and widely spaced teeth [18]. The presence of minimal limb abnormalities has been described in one HJMD patient [6].

Our aim is to present the first Spanish case with HJMD due to mutations in the *CDH3* gene and the importance of an accurate genetic and clinical diagnosis.

## Case presentation

**Fig. 1:**
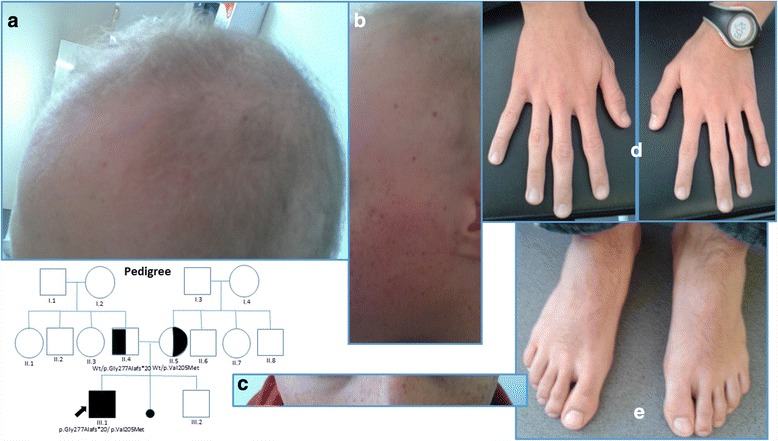
Clinical Genetics’ examination and Pedigree. **a-e**: Clinical Genetics’ examination: pale and dry skin, hypotrichosis of scalp and face (**a-c**). Scalp hair: blond and brittle (impression of pili torti) (A and B). Pigmented lesions in face impressive of keratosis and centro-facial lentigine (B and C). No limbs anomalies (**d** and **e**). Pedigree: Wt = wild type

A male born in 1998, from the north east of Spain was referred to Clinical Genetics with a diagnosis of Keratosis Follicularis Spinulosa Decalvans (OMIM 308800, probable Siemens Syndrome) and Retinitis Pigmentosa Inversa. He was referred in 2011 to the Department of Medical Genetics at the Fundación Jiménez Díaz University Hospital for genetic study. The parents and a nine year old brother were alive and healthy. Nothing of note was reported at a family level, and neither consanguinity nor endogamy existed (Fig. 1. Pedigree).

The Dermatological report stated absence of growing hair since the 1^st^ year of age, pale skin, sparse scalp hair, punctiform follicular keratosis on cheeks, forehead, eyebrows and anterior surface of the arms and posterior surface of the legs, erythema with follicular hyperkeratosis and reduction of hair density on eyebrows.

A skin biopsy had been performed, and was informed as presenting a reduction in the number of scalp follicles, absence of seborrheic glands and infundibular cyst with keratin granules. The suspected diagnosis was Keratosis Follicularis Spinulosa Decalvans (Siemens syndrome).

The patient had been diagnosed with retinal alteration at the age of two (no other data was available). Further ophthalmological examination reports were available for ages four, ten and twelve (Table 1).

**Table 1:** Patient’s ophthalmological examinations since the age of four

Age	Visual Acuity and Visual Field (RE/LE)	Ophthalmoscopy	VEP	Electroretinogram
4years–5years	First symptoms: reduction of VA and central VF. VA: 0.08/0.1 (5years) VF: central scotomae BE	Macular alteration with spicules BE	NA	NA
10year	NA	NA	Abnormal	RE: scotopic: normal, cones response: lower limit of normality. LE: moderate decline of all responses
12years	VA 0.15/0.15. VF: Loss of the central 20° and 10° RE and LE respectively, conserved peripheral field	Macular atrophy with pigment accumulation BE		NA

*BE* Both eyes, *LE* Left eye, *NA* Not Available, *RE* Right eye, *VA* Visual Acuity, *VEP* Visual Evoked Potentials, *VF* Visual Field, *Yr* Years

The patient underwent an excision of a pedunculated colorectal polyp and had been diagnosed with Nodular Lymphoid Hyperplasia of the gastrointestinal tract at the age of two.

The patient was first referred to our Department for genetic testing (reports and sample) in 2011, with a diagnosis of two unrelated diagnosis, Siemens Syndrome and Retinitis Pigmentosa Inversa, for testing of the retinal dystrophy. Further, he was seen at Clinics at the Department of Genetics at the age of 14 and 15 (Table 2), when his diagnosis was re-assessed as likely presenting HJMD, which accounted for both conditions (both the retinal dystrophy and the hypotrichosis).

**Table 2:** Timeline

Year (age in years)	
2000	-First visit to Dermatology due to absence of hair growth -Excision of a pedunculated colorectal polyp: Nodular Lymphoid Hyperplasia of the gastrointestinal tract
2002 (4)	-First visit to Ophthalmolgy: Maculopathy was suspected
2003 (5)	-Maculopathy vs inverse retinitis pigmentosa -Skin biopsy. Suspicion of Keratosis Follicularis Spinulosa Decalvans (Siemens Syndrome)
2005 (7)–2010 (12)	-Ophthalmological follow up
2011 (13)	-Referral for Genetic testing (bloods and reprots): clinical suspicion *ABCA4* –related maculopathy + Keratosis Follicularis Spinulosa Decalvans -Genetic tests: APEX (Arrayed Primer Extension) chip, sequencing and MLPS (multiplex ligation-dependent probe amplification) for *ABCA4* gene: heterozygous for c.6148G > C, p.Val2050Leu. No second allele was found
2012 (14)	-First visit to Clinical Genetics, diagnosis re-assessment: Hypotrichosis with Juvenile Macular Dystrophy
2013 (15)	-Genetic Tests: *CDH3* sequencing: c.830del; p.Gly277Alafs*20 (paternal) and c.613G > A; p.Val205Met (maternal) -2^nd^ visit to Clinical Genetics, confirmed diagnosis: Hypotrichosis with Juvenile Macular Dystrophy due to *CDH3* mutations

Clinical Genetics examination at the age of 14 and 15 revealed generalised pale and dry skin, hypotrichosis that was confined to scalp, face (no incipient beard nor moustache at the age of 16) and arms (Fig. 1 a-e). Scalp hair was blond and brittle (impression of *pili torti*) (Fig. 1a), eyebrows and eyelashes (not shown) appeared normal; although at a younger age a reduction in their density had been described. Pigmented lesions could be observed in face, they were impressive of keratosis and centro-facial lentigine and to our knowledge they were probably sun exposure related (Fig. 1b and c). No dental, hands or feet anomalies were noticed (Fig. 1d and e); when asked, the patient denied needing special dental care. No other findings were manifest after complete clinical examination.

First suspected diagnosis, based on the referral information, was *ABCA4*–related maculopathy + Keratosis Follicularis Spinulosa Decalvans. However, after clinical examination and further evaluation of the reports, the suspected diagnosis was HJMD.

An automated DNA extractor (model BioRobot EZ1; Qiagen, Hilden, Germany) was used for DNA extraction from peripheral blood collected in EDTA tubes in accordance with manufacturer instructions.

For the study of *ABCA4* gene the following techniques were used: a) Genotyping APEX (Arrayed Primer Extension) for *ABCA4* gene (Asper biotech, Tartu, Estonia, http://www.asperbio.com/asper-ophthalmics/stargardt-disease-cone-rod-dystrophy-abca4/stargardt-disease-targeted-mutation-analysis); b) Sanger sequencing of all the coding exons and intronic flanking sequences, and c) MLPA (Multiplex Ligation-dependent Probe Amplification) analysis using a commercial kit (SALSA® MLPA® P151-P152 *ABCA4* probemix, MRC-Holland, Amsterdam, the Netherland) to study *ABCA4* exonic deletions or duplications. b) And c) where analyzed by capillary electrophoresis (*Abiprism 3130*; *Sequencing Analysis v5.2*), performed at our Genetics Department, Fundación Jiménez Díaz University Hospital, Madrid)

For the study of the *CDH3* gene: a Sanger sequencing of all the coding exons and intronic flanking sequences of *CDH3* was performed at Imegen (Instituto de Medicina Genómica, Valencia, Spain).

For confirmation and segregation of the changes found in *CDH3*, Sanger sequencing was performed at our Genetics Department. The brother was not studied as the majority of international guidelines addressing genetic carrier testing in children do not recommend it. However, we did recommend that when he becomes an adult, a carrier testing (for both *ABCA4* and *CDH3*) is performed.

First, and based on the initial clinical suspicion of two different conditions being present in the patient (patient was referred along with the sample for retinal dystrophy testing as presenting two unrelated entities), a study of isolated juvenile macular dystrophy was performed. Only a heterozygous missense mutation (c.6148G > C, p.Val2050Leu; NM_000350 *RefSeq*) in *ABCA4* was found. After a comprehensive analysis of coding regions and gene rearrangements a second allele was not found.

Further, and after the re-assessment of the patient on his appointment at Clinical Genetics, *CDH3* (NM_001793.5 *RefSeq*) sequencing allowed to detect compound heterozygous variants: a novel maternal missense change c.613G > A; p.Val205Met, predicted as probably pathogenic by *in silico* analysis (dataset dbnsfp30a implemented in ANNOVAR was used. (Table 3), and a previously reported paternal frameshift c.830del; p.Gly277Alafs*20 [7].

**Table 3:** Functional prediction of p.Val205Met variant on CDH3 gene. Prediction and score from 12 different predictors

Nucleotide variation: NM_001793.5:c.613G > A Amino-acid variation: p.Val205Met Mutation type: nonsynonymous SNV Exon: 6	
In silico predictors	Prediction (score)
SIFT	Deleterious (0)
Polyphen	Deleterious (1)
Mutation Taster	Deleterious (1)
Align GVGD	Tolerated (Class 0)
PROVEAN	Deleterious (−2.81)
CADD	Deleterious (22.8)
MCAP	Deleterious (0.086)
LRT	Tolerated (0.244)
MutationAssessor	Deleterious (3415)
MetaLR	Deleterious (0.58)
fathmm-MKL	Deleterious (0.994)
MetaSVM	Deleterious (0.301)

**Fig. 2:**
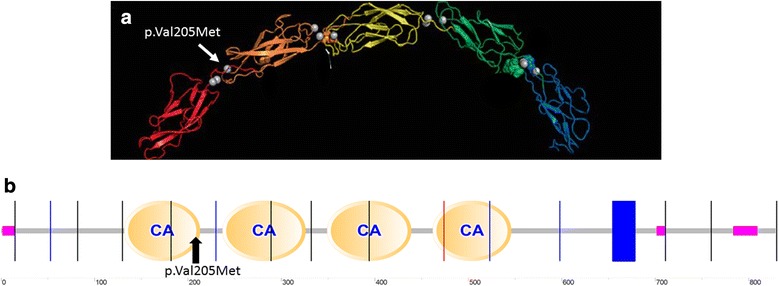
Human cadherin 3 protein structure models. Legend: **a**: Human cadherin 3 protein structure model. The colours (red, orange, yellow green and blue) represent the 5 cadherin domains. The white arrow shows the p.Val205Met mutation position at a calcium-binding domain (grey circles). Image obtained from http://www.proteinmodelportal.org/?pid=modelDetail&provider=SWISSMODEL&template=1l3wA&pmpuid=1000801449293&range_from=1&range_to=829&ref_ac=P22223&mapped_ac=&zid=async (date of access: 04/04/2016). **b**: Human cadherin 3 protein model highlighting calcium binding sites. The back arrow shows the p.Val205Met mutation position. Image obtained form: http://smart.embl.de/smart/show_motifs.pl?ID=P22223 (date of access: 04/04/2016)

Several evidences strongly support that p.Val205Met in *CDH3* is a very likely pathogenic variant and seems to be responsible for our patient’s clinical findings: a) high evolutionary conservation of the amino-acid residue that is located at a calcium-binding site (sites crucial for function and stability of cadherins [25], Fig. 2a and b), b) an in silico damaging effect on the protein is predicted in 10 out of 12 in silico tools, (Table 3), c) its absence in ExAC database (http://exac.broadinstitute.org/gene/ENSG00000062038, date of access 04/04/2016), d) the is change co-segregating with the disease,, and e) a phenotype highly suggestive of *CDH3.*

This way, clinical revision along with directed genetic study allowed reclassifying this patient as HJMD due to *CDH3* mutation.

## Conclusions

We have presented a patient with a phenotype of HJMD. The fact that no other alterations were observed, apart from retinal dystrophy and hypotrichosis, allowed us to discard EEM. Although no radiological examination of hand or feet was performed, which would completely rule out EEM, on clinical examination no abnormality was noted. Mild anomalies of hand and feet have been noted in EEM patients misdiagnosed with HJMD [6, 19], we believe that this is not the case in our patient. Mild changes (as subtle shortening of distal phalanges or mild toe syndactyly) were not observable on the thorough clinical examination, performed on our patient.

The presence of two compound heterozygous mutations in the *CDH3* gene in our patient, supported our clinical suspicion.

The *ABCA4* gene was studied because it is the most frequent gene responsible for macular dystrophy in the young [26] and the retinal phenotype stated in the available reports were suggestive of macular dystrophy. As previously mentioned, first suspected diagnosis, based on the referral information, was *ABCA4*–related maculopathy + Keratosis Follicularis Spinulosa Decalvans. Even if our patient presented a mutation on the *ABCA4* gene, it was discarded as responsible for the patient’s maculopathy as no second mutation was found. We cannot rule out that the p.Val2050Leu mutation in *ABCA4* is playing a role in the patient’s ophthalmological phenotype. However, there is no known functional interaction between *ABCA4* and *CDH3* at a genetic or protein level; and, the pathogenic effect of the p.Val2050Leu change in *ABCA4* is nowadays discussed (http://www.ncbi.nlm.nih.gov/clinvar/?term=%22Retina%20International%22[submitter]+AND+%22ABCA4%22[gene], date of access 04/04/2016). Additionally, the prevalence of patients affected by retinal dystrophy carrying an *ABCA4* mutation with another causative gene for their retinal dystrophy is not rare [27].

As it has previously been noted the main differential diagnostic of HJMD from an ophthalmological point of view is not only Stargardt’s disease, which is caused mainly by *ABCA4* mutations [20], but any retinal dystrophy phenotype suggestive of *CDH3*, regardless of the hair phenotype [22]. However, when patients access a Clinical Genetics Department they have to be evaluated as a whole. The presence of two genetic disorders in a given patient, one being retinal dystrophy may occur [28]. Nevertheless, in these co-occurrence cases, it is more likely that the different signs and symptoms are due to the same genetic cause.

Nowadays, performing an accurate diagnosis is essential since cell and gene therapy are being evaluated in retinal dystrophies [29, 30], and phenotypic and genetic characteristics are to be considered when selecting patients for future treatments [23].

Besides, the suspected dermatological diagnosis (Siemens Syndrome, OMIM: 305100) is an X-linked trait and has additional skin changes, not present in our patient. Therefore, the patient’s pedigree and phenotype were unlikely to suggest this inheritance model and diagnosis.

Interestingly, our patient’s retinal alteration was first noticed when he was two years of age and he was diagnosed as presenting Retinitis Pigmentosa Inversa. This fact supports the idea of an early onset retinal dystrophy rather than a juvenile onset, and the existence of a major implication of the retina beyond the macula [7, 20–23].

Despite the fact that the missense change p.Val205Met in *CDH3* has not been reported previously, we have presented evidence of its pathogenicity. Moreover, a missense change has been described in the same aminoacid position (p.Val205Leu) with a minor allele frequency of 2*10^−5^ in Asian and Latino populations (http://exac.broadinstitute.org/gene/ENSG00000062038, date of access 04/04/2016).

To date, the *CDH3* gene is the only gene known to be responsible for HJMD.

HJMD is a very rare disease, fact that makes both its clinical and genetic diagnosis difficult. However, clinical aspects when taken together should guide us to a clinical diagnosis and lead to us to the specific genetic test to be performed.

The presence of two compound heterozygous mutations in this gene in our patient with a phenotype highly suggestive of HJMD and the absence of additional systemic anomalies led us to the diagnosis of the first Spanish case, and one of the very few Caucasian cases, of HJMD and to describe a new mutation in the *CDH3* gene.

Moreover, not only we prove that an accurate clinical genetic diagnosis is of crucial relevance for leading directed genetic studies, but we have widened the number of both HJMD cases and *CDH3* mutations.

Additionally, we also present the importance of differential diagnosis in retinal dystrophies (syndromic or not) as prognosis, follow up, chance of treatment and familiar implications are deeply linked to the responsible gene.

## Abbreviations

BE: Both eyes
EEM: Ectodermal dysplasia, ectrodactyly and macular dystrophy
HJMD: Hypotrichosis with juvenile macular dystrophy
LE: Left eye
MLPA: multiplex ligation-dependent probe amplification
RD: Retinal dystrophy
RE: Right eye
SHFM: split hand/foot malformation
VA: Visual acuity
VEP: Visual evoked potentials
VF: Visual field
Yr: Years
